# Lymphangiogenesis and lymph node metastasis in breast cancer

**DOI:** 10.1186/1476-4598-7-23

**Published:** 2008-03-06

**Authors:** Giles H Cunnick, Wen G Jiang, Tony Douglas-Jones, Gareth Watkins, Kelvin F Gomez, Mike J Morgan, Ashok Subramanian, Kefah Mokbel, Robert E Mansel

**Affiliations:** 1 Metastasis and Angiogenesis Research Group, Cardiff University School of Medicine, Cardiff, CF14 4XN, Wales, UK; 2 Dept of Histopathology, Cardiff University School of Medicine, Cardiff, CF14 4XN, Wales, UK; 3 Department of breast surgery, St. George's Hospital, Tooting, London, SW17 0QT, England, UK

## Abstract

**Introduction:**

There have been few studies on lymphangiogenesis in the past due to the lack of specific lymphatic endothelial markers, and lymphatic-specific growth factors. Recently, these limitations have been relieved by the discovery of a small number of potential lymphatic-specific markers. The relationship between lymphangiogenesis and regional or distant metastasis has not previously been investigated in humans. Using these lymphatic markers, it is possible to explore the relationship between lymphangiogenesis and tumour metastasis. This study indirectly quantified lymphangiogenesis by measuring mRNA expression of all seven lymphatic markers described above in breast cancers and correlated these markers with lymphatic involvement and survival.

The cDNA from 153 frozen archived breast samples were analysed with Q-PCR for all seven lymphangiogenic markers. This was correlated with various prognostic factors as well as patient survival.

**Results:**

There was significantly greater expression of all 7 markers in malignant compared to benign breast tissue. In addition, there was greater expression in lymph node positive/grade 3 tumours when compared to lymph node negative/grade 1 tumours. In 5 of the markers, there was a greater expression in poor NPI prognostic tumours when compared to favourable prognostic tumours which was not statistically significant. There was no association between recurrence risk and lymphangiogenic marker expression.

**Conclusion:**

In summary, the findings from this study show that lymphangiogenesis, measured by specific lymphatic marker expression, is higher in breast cancers than in normal breast tissue. Secondly, breast cancers which have metastasised to the regional lymphatics show higher expression compared to those which have not, although the individual differences for all five markers were not statistically significant.

## Introduction

Breast cancer is one of the leading causes of cancer death in the female population in the Western World affecting as many as one in ten women in the UK [1], and its incidence appears to be rising. Although earlier diagnosis and better treatment are now available, many of the mechanisms underlying its ability to metastasise are poorly understood. Breast cancer spreads primarily via the lymphatic system. Regional lymph nodes are usually the first metastatic sites to be involved, often followed by distant metastasis to the lungs, liver and bones. Although various prognostic factors are known, regional lymph node status is the single most important prognostic factor in breast cancer; patients with axillary metastasis at the time of diagnosis have a much worse prognosis than those without metastasis [2,3].

Clinical and pathological observations have long suggested that for many other tumours, the most common pathway of initial dissemination is also via lymphatics, with patterns of spread via afferent vessels following routes of natural lymphatic drainage. However, the lymphatic system has traditionally been overshadowed by the greater emphasis placed on angiogenesis, the formation of new blood vessels. Indeed, it is widely accepted that angiogenesis is necessary for the growth and metastatic spread of solid tumours [4,5]. There have been relatively few studies on lymphangiogenesis in the past due to the lack of suitable markers that distinguish lymphatic from blood vascular endothelium, and the lack of lymphatic-specific growth factors. Furthermore, it is not known whether pre-existing lymphatic vessels are sufficient to permit initial tumour metastasis, or whether tumour dissemination requires the development of new lymphatics [4].

In recent years, these limitations have been relieved by the discovery of a small number of potential lymphatic-specific markers [5]. These include: LYVE-1, a lymphatic endothelial receptor for hyaluronan [6], Prox1, a homeobox gene product involved in regulating early lymphatic development [7], podoplanin, a glomerular podocyte membrane mucoprotein which is also found on lymphatic endothelium, but not in blood vessels [8], 5'-nucleotidase, an enzyme whose activity is very high in the lymphatic capillaries, but much lower in the blood capillaries [9], and the vascular endothelial growth receptor-3 (VEGFR-3) which is a transmembrane tyrosine kinase receptor predominantly expressed on the lymphatic endothelium [10]. VEGFR-3 has been shown to control the development and growth of the lymphatic system [11] VEGF-C [12] and VEGF-D [13] are two polypeptide growth factors which are agonists of the VEGFR-3 receptor, and may therefore be considered to be lymphangiogenic [14] These factors have been shown to be associated with lymphatic and distant metastasis and shorter overall survival [15]. In contrast, the angiogenic growth factor, VEGF, does not bind to VEGFR-3.

Breast cancer angiogenesis has been clearly linked to tumour metastasis. Using histopathological staining methods for blood vessel endothelial markers, a significant direct correlation was found between the highest microvessel density in histological sections of human invasive breast cancer and the occurrence of metastases [16]. Notwithstanding these findings, no studies have shown that angiogenesis is correlated with regional lymph node metastasis in breast cancer. The relationship between lymphangiogenesis and regional or distant metastasis has not previously been investigated in humans. Real-time quantitative polymerase chain reaction (QPCR) has now become an established method of quantifying genetic sequences [17]. Using QPCR, a novel approach for quantifying lymphatic markers has been recently described in breast cancer, using LYVE-1 [18].

Using these new lymphatic markers, it is possible to explore the relationship between lymphangiogenesis and tumour metastasis. The aim of this study was to indirectly quantify lymphangiogenesis by measuring mRNA expression of all seven lymphatic markers described above in breast cancers and correlate these markers with lymphatic involvement and survival.

## Methods

### Specimens

153 frozen archived breast samples from 105 patients were kept at -80°C. The samples consisted of breast cancers and background benign breast tissue. Histopathological information and patient follow-up details of the specimens were collected but blinded until the end of the study. 7 μm sections were cut from each specimen for histopathology (see below) and ten adjacent 10 μm sections were stored for subsequent RNA extraction. Human umbilical vein endothelial cells (HUVECs), a fibroblast cell line (MRC-5), and two breast cancer cell lines (MB MDA 231 and MCF7), were also cultured.

### Histopathology

All samples were stained with haematoxylin and eosin for routine histopathological assessment by a consultant pathologist to confirm whether or not tumour was present in the sample. The percentage of tumour, with respect to surrounding stroma, was also estimated. Each assessment was later compared with the patients' original histopathological reports. In addition, sections were also stained with factor 8 monoclonal antibodies, using standard immunohistochemical techniques. Five microscope fields were counted for each case and summated to give a single figure. Ten sections were blindly counted a second time to exclude the presence of intra-observer error.

### RNA Extraction and cDNA Synthesis

RNA was extracted from the homogenised breast samples and cell lines using the standard RNAzol procedure. The concentration of RNA was measured with a UV spectrophotometer. Equal amounts of cDNA were subsequently synthesised from 1 μg of total RNA using a reverse transcription kit (Abgene, Surrey, UK) according to the manufacturer's instructions.

### PCR

A pair of primers specific for part of the following molecules were designed, using the Beacon Designer software (Palo Alto, California), which were based on cDNA sequences obtained from the Gene Bank: LYVE-1, Prox1, podoplanin, 5'-nucleotidase, VEGF-C, VEGF-D, VEGFR-3, VEGFR-2 and β-actin. The forward and reverse primer sequences are shown in Table 1.

**Table 1:** Primers used for PCR analysis

Molecule	PCR Primer
LYVE:1	5'-gtgcttcagcctggtgttg-3' 5'-gcttggactcttggactcttc-3'
Prox-1	5'-atgtcatctcaccacctgag-3' 5'-gcagatgaccttgtatatgg-3'
Podoplanin	5'-tggtggaatcatcgttgtggttatgc-3' 5'-acaagtgaacctcctgcctcctc-3'
5'-Nucleotidase	5'-tggagtgatcggttacagaatgc-3' 5'-gctggtgccgctgtgagtc-3'
VEGF-C	5'-ggcttctcctggtgacatctg-3' 5'-ttgcttgggacacattgacattc-3'
VEGF-D	5'-ctaatgtcaactgcttag-3' 5'-atggtctggtatgaaagg-3'
VEGFR-3	5'-gcgactgtggctctg-3' 5'-gaaaggaagtcctggtctc-3'
VEGFR-2	5'-gcctctgtgggtttgcctagtgtttc-3' 5'-gctgatcatgtagctgggaat-3'
β-actin	5'-atgatatcgccgcgctcgt-3' 5'-cgctcggtgaggatcttca-3'

Please note for all tables, the number in parentheses gives the number of samples valid for analysis following PCR.

Conventional PCR was also performed using cDNA of HUVECs, MRC-5, MDA-MB-231 and MCF7 using all nine pairs of primers. Electrophoresis of the PCR products was performed on a 0.8% agarose gel and stained with ethidium bromide prior to examination under UV light. Quantitation of mRNA was performed by normalisation vs CK-19.

### Plasmid Synthesis

The appropriate PCR products were TA cloned using the PCR^®^2.1-TOPO vector and amplified using One-Shot E. coli (Invitrogen, Groningen, Netherlands) following the manufacturer's instructions. X-gal in LB agar plates was used to identify the positive colonies. The positive colonies were further verified by PCR, using the same corresponding pairs of primers. The plasmids were then extracted from the E. coli by using the plasmid mini purification kit (Qiagen, Crawley, UK) according to the manufacturer's guidelines. The plasmids were digested using the *Hind III *and *Xba I *restriction endonucleases to confirm the presence of the inserts. In addition the plasmids were sequenced to confirm that they contained the correct DNA sequences (BigDye, PE Applied Biosystems, Warrington, UK). The concentration of each plasmid was calculated and serial logarithmic dilutions were prepared.

### Real-time quantitative polymerase chain reaction (QPCR)

The detection of LYVE-1 expression used a Scorpions system. The Scorpions probe/primer was formed by linking a forward primer and fluorescent probe using a PCR stopper (Oswel, Southampton, UK) [19]. Taqman primers and probes were designed for VEGFR-3 and VEGFR-2. For the other five markers, Prox1, podoplanin, 5'-nucleotidase, VEGF-C and VEGF-D, the Amplifluor system [20] was used required the use of pairs of specific primers for each marker (a forward primer and reverse Z primer), with a universal probe (UniPrimer™) which was used for all five markers. Using the iCycler IQ system (BioRad, Camberley, UK), which incorporates a gradient thermocycler and a 96 channel optical unit, the plasmid standards and breast cancer cDNA were simultaneously assayed. RTQPCR conditions were as follows: Scorpions: Denaturing: 95°C for 15s; Annealing: 54°C for 20s; Extension: 60°C for 40s. Taqman: 95°C for 15s, 54°C for 20s, 60°C for 40s. Amplifluor: 95°C for 15s, 55°C for 60s, 72°C for 20s. Using the purified plasmids as internal standards, the level cDNA (copies/μl) of each of the seven markers in the breast samples were calculated. Negative template controls were included in during all RTQPCR procedures. The size of the products of RTQPCR were subsequently verified on agarose gels. Primer sequences are shown in Table 2.

**Table 2:** Primers used for Q-PCR analysis

Molecule	Q-PCR Primer
LYVE-1	5'-FAM-CCGCGGGATGGAAAGCTCTTCTGCCGCGG-MR-HEG-GGTCCAAGGCTCTTTGCGT-3' 5'-AAATTCAGCTGCTGGTTCGC-3'
VEGFR-3	5'-ACGGCCTGGTGAGTGGC-3'(Forward) 5'-CGTTTGACTCCTCCGTGATG-3' (Reverse) 5'-FAM-CCATGACCCCCC CGACCTTGA-3' [53]
VEGFR-2	5'-TGTGGCTCTGCGTGGAGA-3' (Forward) 5'-GGGCAGATCAAGAGAAACACTAGG-3' (Reverse) 5'-FAM-CGGGCCGCCTCTGCGGGTTT-3' [54]
Prox-1	5'-GCAGGAAAAGTTCTACCA-3' (Forward) Z: 5'-TCTTCAGACAGGTTACCATC-3' (Reverse)
Podoplanin	5'-GAATCATCGTTGTGGTTATG-3' (Forward) Z: 5'-CTTTCATTTGCCTATCACAT-3' (Reverse)
5'nucleotidase	5'-tcgacctcctggagtgatcgg-3' (Forward) Z: 5'-cacccgatgataggcttctc-3' (Reverse)
VEGF-C	Z: 5'-GCTTCTCCTGGTGACATC-3' (Forward) 5'-CCTGAGTCCCTCGTCTCT-3' (Reverse)
VEGF-D	5'-GCTCCAGTAATGAACATGG-3' (Forward) Z: 5'-ATCTGCTGTTCAGATCGTT-3' (Reverse)

Please note for all tables, the number in parentheses gives the number of samples valid for analysis following PCR.

### Statistical Analysis

After unblinding the results, mean levels of each marker were compared in lymph node positive patients with lymph node negative patients and background normal breast tissue. Marker levels were also compared between patients with good, moderate or poor prognosis tumours, using the Nottingham Prognostic Index (NPI), and between patients who were disease-free on follow-up and those with recurrence or who had died. Statistical significance was calculated using the Mann-Whitney U-test.

## Results

### Breast specimen histology and patient details

153 breast samples were analysed. Histopathological examination confirmed that there were 120 breast cancers and 33 normal background breast tissue samples. The percentage of tumour in each sample varied from 2.5% to 100% (mean 48.0; sd 27.8). Using factor 8 immunohistochemical staining, the mean vessel counts for the breast cancers and normal breast tissue were 45.7 ± 23.2 and 51.7 ± 21.0, respectively (t-test: *P *= 0.116).

### Expression of lymphatic markers in cell lines and tissues

A comparison was made of the expression of β-actin and the eight markers in the following cell lines: HUVECs, MRC-5, MDA-MB-231 and MCF7. LYVE-1, Prox1, podoplanin, 5'-nucleotidase, VEGFR-2 and VEGFR-3 were expressed in HUVECs. In addition, Prox1 and 5'-nucleotidase were expressed in MDA-MB-231 cells and VEGFR-2 was weakly expressed in MRC-5 cells. In contrast, VEGF-C and VEGF-D were not expressed in HUVECs, but were both expressed in the aggressive breast cancer cell line, MDA-MB-231. In addition, VEGF-C was expressed in MRC-5 fibroblast cells and VEGF-D was expressed in the MCF7 breast cancer cell line.

### Quantitation of markers in normal breast tissue and breast cancer

153 breast samples were analysed. Gel electrophoresis of all QPCR products of the different markers confirmed that the products were of the correct size. It was found that the mean expression levels for breast cancer were statistically significantly higher than normal breast tissue for all seven lymphatic markers (Table 3). There was no significant difference in VEGFR-2 expression between the two groups.

**Table 3:** Levels of mRNA transcript in tumour vs normal tissue

	**TUMOUR**	**NORMAL BREAST**	***P*-VALUE**
**LYVE-1**	57.0 ± 326.3 01 (93)	0.28 ± 0.37 (23)	< 0.0001
**Prox1**	5099.1 ± 8353.1 (119)	1270.2 ± 1555.4 (32)	0.0009
**Podoplanin**	13722 ± 67277 (120)	4973 ± 7115 (29)	0.0483
**5'-Nucleotidase**	4964.4 ± 7166.3 (112)	3208.1 ± 8760.9 (32)	0.0068
**VEGFR-3**	121.76 ± 223.84 (120)	65.64 ± 197.00 (32)	0.0104
**VEGF-C**	1899.2 ± 8596.4 (84)	270.4 ± 305.5 (23)	0.0255
**VEGF-D**	57.3 ± 113.1 (58)	11.7 ± 12.2 (15)	0.0163
**VEGFR-2**	5.87 ± 16.17 (116)	4.01 ± 12.39 (31)	0.9017
**VEGF-C:VEGF-D ratio**	137.4 ± 60.1	160 ± 111	0.3694

Please note for all tables, the number in parentheses gives the number of samples valid for analysis following PCR.

### Quantitation of markers in lymph node positive and negative breast cancer

It was found that mean expression levels were higher in tumours which had metastasised to the axillary lymph nodes (node positive) than in tumours which had not metastasised (node negative) for all five lymphatic endothelial markers and the two growth factors (Table 4). However, individually, none of these differences reached statistical significance. Expression of VEGFR-2 was higher in node negative tumours, but this difference was not significant. It has recently reported that the VEGF-C:VEGF-D ratio may be a better prognostic marker [21,22]. Here we also examined the VEGF-C/VEGF-D ratio, which proved to be a better marker in distinguishing node negative from node positive tumours (ratio being 317 ± 167 for node positive and 45 ± 19 for node negative tumours, p = 0.0369, Table 5).

**Table 4:** Level of mRNA transcript in node +ve vs node -ve tumours

	**Node +ve**	**Node -vE**	**P-Value**
**LYVE-1**	128.0 ± 231.9 (36)	12.1 ± 18.2 (57)	0.89
**Prox-1**	6140.0 ± 10922.0 10571	4466.0 ± 6309.0 (74)	0.82
**Podoplanin**	23306.0 ± 105711.0 (46)	7764.0 ± 19895.0 (74)	0.125
**5'Nucleotidase**	5170.4 ± 6676.5 (42)	4840.8 ± 7489.2 (70)	0.550
**VEGFR-3**	129.4 ± 238.3 (46)	117.0 ± 215.9 (74)	0.480
**VEGF-C**	3989.0 ± 13759.0(32)	613.1 ± 877.4 (52)	0.622
**VEGF-D**	114.5 ± 186.7 1	31.5 ± 36.1 (40)	0.286
**VEGFR-2**	4.2 ± 14.8 (43)	6.8 ± 17.0 (73)	0.570
**VEGF-C:VEGF-D ratio**	317.0 ± 167.0	45.0 ± 19.0	0.0369

Please note for all tables, the number in parentheses gives the number of samples valid for analysis following PCR.

**Table 5:** Levels of mRNA transcript vs tumour grade

	**Grade 1**	**Grade 2**	**Grade 3**
**LYVE-1**	12.3 ± 30.2 (20)	67.0 ± 328.4 (33)	70.9 ± 401.2 (40)
**Prox1**	7083 ± 9542 (24)	2727 ± 4693 (41)	5536 ± 9999 (55)
**Podoplanin**	3099 ± 3579 (24)	12774 ± 26291 (41)	18775 ± 95973 (56)
**5'-Nucleotidase**	5187 ± 7647 (22)	4652 ± 7725 (38)	5615 ± 10888 (52)
**VEGFR-3**	110 ± 161 (24)	122 ± 206 (41)	127 ± 259 (56)
**VEGF-C**	702 ± 270 (15)	1664 ± 942 (29)	2290 ± 1768 (42)
**VEGF-D**	44.3 ± 17.1 (14)	26.2 ± 6.23 (17)	62.6 ± 26.3 (29)
**VEGFR-2**	15.9 ± 30.5 (24)	5.1 ± 11.2 (39)	1.8 ± 3.4 (54)
**VEGF-C/VEGF-D ratioR-2**	160 ± 130	236 ± 176	65.9 ± 26.5

Please note for all tables, the number in parentheses gives the number of samples valid for analysis following PCR.

### Correlations with tumour size, ER status, grade and type, NPI and survival

Although most of the differences for each marker failed to reach statistical significance, the trend was for grade three tumours to have the highest marker expression and grade one tumours to have the lowest expression (Table 6). Marker expression was compared between the two commonest tumour types, ductal and lobular adenocarcinoma (Table 7) with no consistent pattern of expression observed. In five out of the six lymphatic markers, expression was found to be highest in the poor NPI prognosis group, although most of the differences were not statistically significant (Table 8). Neither tumour size nor ER positivity (Table 9) was correlated with marker expression. Finally, after comparing expression levels between tumours in which the patients were alive and well on clinical follow-up and those who had had recurrence of their breast cancer (Table 7), we did not find any consistent pattern between the groups after a median follow-up of 72 months. None of the comparisons were significant (Mann-Whitney > 0.05 for all markers).

**Table 6:** Levels of mRNA transcript vs tumour type

	**Ductal Tumours**	**Lobular tumours**	**P value**
LYVE-1	42.4 ± 295.4 (74)	241.1 ± 666.3 (8)	n/s
PROX-1	5743.0 ± 9206.0 (97)	2928.0 ± 4117 (10)	n/s
PODOPLANIN	15498.0 ± 74049.0 (98)	1783.0 ± 2657.0 (10)	n/s
5'-nucleotidase	4813.0 ± 6873.0 (91)	1522 ± 2371 (10)	n/s
VEGFR-3	126.3 ± 239.4 (98)	99.0 ± 149.3 (10)	n/s
VEGF-C	2098 ± 1093 (72)	524 ± 334 (3)	n/s
VEGF-D	47.3 ± 15.8 (49)	78.6 ± 45.1 (5)	n/s
VEGFR-2	4.2 ± 14.7 (94)	10.1 ± 14.5 (10)	n/s
VEGF-C/VEEGF-D ratio	119.0 ± 65.5	293.0 ± 258.0	n/s

Please note for all tables, the number in parentheses gives the number of samples valid for analysis following PCR.

**Table 7:** Levels of mRNA transcript vs prognosis

	**GOOD**	**MODERATE**	**POOR**
**LYVE-1**	13.8 ± 34.3 (35)	3.4 ± 6.6 (37)	272.9 ± 140.9 (17)
**PROX-1**	4785.0 ± 7723.0 (44)	4354.0 ± 4530.0 (49)	7638.0 ± 14562.0 (22)
**PODOPLANIN**	4341.0 ± 4919 (44)	10353.0 ± 22574 (49)	37811.0 ± 149439 (23)
**5'-Nucleotidase**	4708.0 ± 8053 (40)	44217.0 ± 4337 (45)	6471.0 ± 15237.0 (22)
**VEGFR-3**	117.0 ± 171.0 (44)	122.0 ± 252 (49)	133.0 ± 268.0 (23)
**VEGF-C**	467.0 ± 665.0 (45)	911.0 ± 1187.0 389.0(	3989.0 ± 13759.0 (32)
**VEGF-D**	29.3 ± 28.8 (36)	117.3 ± 198.9 (15)	83.7 ± 106.6 (6)
**VEGFR-2**	12.2 ± 25.0 (43)	2.1 ± 4.0 (48)	2.4 ± 4.3 (21)
**VEGF-C:VEGF-D**	45.0 ± 19.0	412.0 ± 77.0 (73)	77.0 ± 73.0

Please note for all tables, the number in parentheses gives the number of samples valid for analysis following PCR.

**Table 8:** Levels of mRNA transcription vs outcome

	**ALIVE AND WELL**	**RECURRENCE**	**p-VALUE**
**LYVE-1**	79.1 ± 389.0 (65)	6.1 ± 11.0 (23)	0.932
**PROX-1**	4568.0 ± 6729.0 (84)	7420.0 ± 12336 (31)	0.076
**Podoplanin**	13605.0 ± 78024 6550	9336.0 ± 15008.0 (31)	0.186
**5'Nuceotidase**	4230.0 ± 6550.0 (78)	8091.0 ± 14398.0 (29)	0.203
**VEGFR-3**	113.0 ± 229.0 (85)	149.0 ± 227.0 (31)	0.064
**VEFG-C**	1124.0 ± 464.0 (60)	3919.0 ± 3370.0 (22)	0.660
**VEGF-D**	47.9 ± 17.1(44)	60.3 ± 24.5 (12)	0.881
**VEGFR-2**	5.5 ± 15.2 (83)	4.1 ± 99.0 (29)	0.375
**VEGFC:VEGF-D ratio**	148.0 ± 78.0	108.0 ± 106.0	0.77

Please note for all tables, the number in parentheses gives the number of samples valid for analysis following PCR.

**Table 9:** Levels of mRNA transcripts ve Oestrogen receptor (ER) status

	ER(-)	ER(+)	P value
LYVE1	53.6 ± 10.1	66.7 ± 17.9	0.53
PROX1	6394 ± 880	2860 ± 2429	0.57
Podoplanin	16923 ± 11842	5664 ± 1255	0.24
5-Nucleotidase	4358 ± 716	7544 ± 2458	0.22
VEGF-R3	111 ± 27	144 ± 39	0.47
VEGF-C	1189 ± 507	3748 ± 3222	0.44
VEGF-D	50 ± 19	33.5 ± 18	0.53
VEGF-R2	5.2 ± 1.9	6.0 ± 1.8	0.76
VEGF-C/VEGF-D ratio	128.6 ± 81.8	93.524136.6	0.70

Please note for all tables, the number in parentheses gives the number of samples valid for analysis following PCR.

## Discussion

This is the first attempt to quantify lymphangiogenesis in tissues from a cohort of cancer patients. Moreover, we have assayed all currently known lymphatic markers, as well as an angiogenic marker. It was found that all lymphatic endothelial markers, LYVE-1, Prox1, podoplanin, 5'-nucleotidase and VEGFR-3, were expressed in significantly higher levels in breast cancers than in normal breast tissue. Since this indirectly reflects the rate of synthesis of lymphatics, this confirms for the first time that lymphangiogenesis is greater in breast cancer than it is in normal breast tissue. This contrasts with the historical view that tumours do not contain any functional lymphatic system [21]. Secondly, lymphangiogenesis appears greater in tumours which have metastasised to the regional lymph nodes than in tumours which have not metastasised. Although the differences between node positive and negative tumours were not statistically significant for individual markers, all five markers expressed higher levels in the node positive breast cancers. In addition, the VEGF-C:VEGF-D ratio ratio however was significantly higher in the node node +ve group which concurs with previous studies [21,22] which link this relationship to poor prognosis. This suggests that a real difference may exist, although larger sample numbers are required to confirm this. Recent studies showing significantly increased lymphangiogenesis in and around metastatic lymph nodes have supported this hypothesis [22] which may be present before metastasis actually occurs [23,24] and may be related to VEGF-C expressing tumour associated macrophages [25]

Factor 8 R:Ag has proven to be one of the best available immunohistochemical markers for the identification of endothelial cells [26]. Factor 8 immunohistochemistry is a well-established method of identifying the vessel density in histological specimens. We did not demonstrate any significant difference in the level of factor 8 staining between normal breast tissue and breast cancer tissue. This suggests that the vasculature of the normal breast is similar in density to breast cancer which supports the observation that VEGFR-2 expression was not significantly different between breast tumours and normal breast tissue. It is interesting however, that other groups have found a high microvessel density, measured by factor 8 immunohistochemistry, was associated with a high incidence of metastasis (axillary or distant or both) in breast cancer [27] and also that regional lymph node lymph vessel density was positively correlated with lymph node metastasis, VEGF-C expression and a poor prognosis [28,29]

VEGF-C and VEGF-D are predominantly expressed in human adult tissues, including heart, muscle, ovary and small intestine, but they have also been identified in several types of malignant tumours [30]. It is known that VEGF-C is synthesised as a prepropeptide which undergoes proteolytic maturation to a varying degree [31]. These shorter peptides bind to VEGFR-3, but only the fully processed form can bind to VEGFR-2. Overexpression of VEGF-C in the skin or pancreas of transgenic mice resulted in lymphatic endothelial proliferation and vessel enlargement, while the blood vessels remained unaffected [32]. Furthermore, others have shown that there is a strong association between VEGF-C expression and microlymphatic vessel density in human malignant mesotheliomas [33]. In view of these findings, it is thought that VEGF-C is predominantly a regulator of lymphangiogenesis, although it also does promote angiogenesis to a lesser degree. Since VEGF-D possesses a similar structure with a comparable expression pattern, it is also thought that this growth factor behaves similarly. In addition to promoting lymphangiogenesis, it has now been shown that VEGF-D promotes lymphatic metastasis in a mice [34]. Likewise, using human breast cancers transplanted onto mice, VEGF-C has also been shown to potently increase lymphangiogenesis and promote metastasis to regional lymph nodes [35,36]. We have now shown in this study that expression of VEGF-C and VEGF-D is higher in lymph node positive than negative tumours, although the differences were not statistically significant.

LYVE-1 was first described in 1999, and is an important hyaluronan receptor which is found on the lymph vessel wall and related to the CD44 receptor, although its precise function is unclear. It is completely absent from blood vessels and, therefore, specific for lymphatics. Recent mouse studies have used LYVE-1 in this context. The homeobox gene Prox1 was first described in 1993. Analysis of the expression pattern suggested that it has a functional role in a variety of tissues, including lens, heart, liver, pancreas and central nervous system. It has now also been shown that Prox1 is expressed in a subpopulation of endothelial cells that gives rise to the lymphatic system in mice and is thought to be a specific and required regulator for the development of the murine lymphatic system [37]. It is also absent from blood vessels, making it a useful lymphatic marker. Since the discovery of podoplanin in 1997 [38], expression of this protein has been found in the endothelium of lymphatic capillaries, but not in the blood vasculature. In purely lymphatic tumours (lymphangiomas and hygromas), high levels of podoplanin staining were found, whereas in purely vascular tumours, much lower levels of staining were found when compared to other known vascular endothelial markers. Antibodies to podoplanin have recently been used to demonstrate that high lymphatic microvessel density is associated with lymph node metastasis in human breast cancer [39]. VEGFR-3 is predominantly expressed on lymphatic endothelium, but although previous studies have used this receptor as a specific marker for lymphatic vessels, it has now been shown to be expressed in tumour blood vessels during neovascularisation [40] and so cannot be considered entirely specific for lymphatics. 5'-nucleotidase is an enzyme found throughout the body, however, its activity is significantly higher in lymphatics than blood vessels [41]. For this reason, enzyme histochemistry has been used in the past in order to identify lymphatics by the light and scanning electron microscopes. The expression of this enzyme has not previously been investigated in humans.

In this study therefore, it has been shown that expression of those factors which have been shown to play an active role in lymphangiogenesis is increased in malignant human breast tissue versus its benign counterpart. This is true both for the lymphangiogenic agonists (VEGF-D/C) and their receptors (VEGFR-2/3) but also for the independent markers of lymphatic activity (LYVE-1, Prox-1, podoplanin and 5' nucleotidase). It has also been shown that the VEGF-C:D ratio may have important prognostic significance of it's own.

In this study, we found that all known endothelial markers, LYVE-1, Prox1, podoplanin, 5'-nucleotidase, VEGFR-3 and VEGFR-2 were expressed by the HUVECs. To date, there are no reports describing isolation of endothelial cells from lymphatic capillaries. HUVECs were used because these cells are foetally-derived are therefore likely to be at an early stage of development and differentiation. It would be expected, therefore, that these cells will express both angiogenic and lymphangiogenic markers. Furthermore, HUVECs have been previously shown to express lymphatic markers, such as LYVE-1, in other studies [42]. Both VEGF-C and VEGF-D were expressed in the breast cancer cell lines. The expression of Prox1 and 5'-nucleotidase by MDA-MB-231 cells is interesting, possibly explained by the poor differentiation of these cancer cells. However, it suggests that Prox1 is less specific than originally thought.

The Nottingham Prognostic Index (NPI) for primary breast cancer was first described in 1982 [43]. It comprises a scoring system based on three variables of the primary tumour, namely tumour size, grade and lymph node status. Using this system, scores are classified into three prognostic groups – good, moderate and poor. The 15-year survivals of these groups are 80%, 42% and 13%, respectively [44]. In this study we did not find any statistical evidence of a correlation between a tumour prognosis and the expression of lymphatic markers.

After following-up the breast cancer patients after a median interval of 72 months, we did not find any difference in any marker expression levels between patients who were still in remission from their original cancer and patients who had had recurrence, either local or distant. We believe the interval of 6 years to be insufficient to show whether any difference does exist or not, since the number of statistical events was very low.

No correlation was found between lymphatic marker expression and either tumour grade or histopathological type. High grade tumours carry a worse prognosis than low grade tumours. Since this study indicates that lymphangiogenesis and angiogenesis are unaffected by grade, the different prognoses are not likely to be due to differences in lymphatic or blood vessel synthesis. The only two histopathological types analysed in this study were invasive ductal adenocarcinoma and invasive lobular adenocarcinoma. Although other types were seen, the numbers of each were too small for statistical analysis. This study indicated that the rate of lymphangiogenesis and angiogenesis was similar in both of the main tumour types.

## Conclusion

In summary, the findings from this study show that lymphangiogenesis, measured by specific lymphatic marker expression, is higher in breast cancers than in normal breast tissue. Secondly, breast cancers which have metastasised to the regional lymphatics show higher expression compared to those which have not, although the individual differences for all five markers were not statistically significant The VEGF-C:VEGF-D ratio ratio however was significantly higher in the node node +ve group which concurs with previous studies [21,22] which link this relationship to poor prognosis. Finally, expression of the lymphangiogenic factors, VEGF-C and VEGF-D were also higher in cancers which had metastasised than those which had not, but again differences were not significant. The potential therapeutic possibilities of targeting lymphangiogenic factors in breast cancer using VEGFR-3 antagonists [45,46], COX-2 antagonists [47], IL-7 antagonists [48] and others have been the subject of recent intense investigation [49]. This may be of particular importance in young premenopausal patients with poorly differentiated [50] or inflammatory cancers [51,52]. Although our findings do not statistically support the hypothesis that lymphangiogenesis is correlated with lymphatic metastasis we feel that larger studies are needed to assess this further.

## Abbreviations

DNA: Deoxyribonucleic acid, RNA: Ribonucleic acid, PCR: polymerase chain reaction, VEGF(R): vascular endothelial growth factor (receptor).

## Authors' contributions

GHC performed PCR/RT-PCR analysis and preparation of the manuscript. WGJ conceived the idea, supervised PCR/RT-PCR analysis and edited of manuscript.

AD-J performed the histopathological analysis and tumour preparation. GW aided in preparing tissue for histopathological analysis and optimising assays. KFG performed an extensive literature review and collected tumour tissue for analysis. MM optomised tissue for histopathological analysis. AS performed extensive editing and preparation of manuscript. KM performed tissue collection and statistical analysis. REM supervised experimental work and manuscript editing. All authors read and approved the final manuscript
